# The cost-benefit of newborn screening for X-linked agammaglobulinemia and related B-cell lymphopenia

**DOI:** 10.1007/s12026-026-09842-7

**Published:** 2026-09-15

**Authors:** Eyal Grunebaum, Vanessa Tenembaum, Jessica Quinn, Fred Modell, Raz Somech

**Affiliations:** 1 The Division of Immunology and Allergy, The Hospital for Sick Children, Toronto, ON Canada; 2 Developmental and Stem Cell Biology Program, The Hospital for Sick Children, Toronto, ON Canada; 3 Jeffrey Modell Foundation, 780 Third Avenue, 47th Floor, New York City, NY 10017 USA; 4 Pediatric Department and the Immunology Service, Jeffrey Modell Foundation Center, “Edmond and Lily Safra” Children’s Hospital, Sheba Medical Center, Tel Hashomer, 52621 Israel; 5 Gray Faculty of Medical and Health Sciences, Tel Aviv University, Tel Aviv, Israel

**Keywords:** Newborn screening, Cost-benefits, KREC, BTK, X-linked agammaglobulinemia, B cell lymphopenia

## Abstract

Early detection of X-linked agammaglobulinemia (XLA) and other profound B cell lymphopenias (BCL) in newborns is crucial for timely intervention and improved clinical outcomes. The kappa light chain rearrangement excision circles (KREC) assay has emerged as an effective specific and sensitive tool for newborn screening (NBS) of XLA and other BCL. Large-scale studies have demonstrated that KREC-based NBS identifies affected infants before symptom onset, enabling prompt initiation of immunoglobulin replacement therapy (IGRT) and prophylactic antibiotics, thereby preventing recurrent infections, chronic organ damage, and reducing healthcare burden. Here, we evaluate the possible cost-benefit of NBS for XLA and other BCL. Economic evaluation shows that adding KREC screening to existing NBS programs is likely to be cost-effective and fulfills established criteria for NBS inclusion, supporting its adoption in universal NBS protocols.

Newborn screening (NBS), first introduced by Dr. Guthrie for Phenylketonuria [1, 2] has revolutionized the ability to diagnose, treat and prevent unfavorable outcomes for many inborn conditions. Affected infants diagnosed through NBS can receive appropriate treatment shortly after birth and before symptoms appear, thereby preventing further damage. Later on, Wilson and Junger proposed criteria for which disease should be considered for NBS [3]. These criteria include (i) There should be a recognizable latent or early symptomatic stage; (ii) The natural history of the condition, including development from latent to declared disease, should be adequately understood; (iii) There should be a suitable test or examination; (iv) There should be an accepted treatment for patients with recognized disease; (v) The cost of case-finding (including diagnosis and treatment of patients diagnosed) should be economically balanced in relation to possible expenditure on medical care.

Since 2008, NBS for profound T cell lymphopenia (TCL), such as severe combined immunodeficiency (SCID), has been implemented in many countries [4]. SCID is among the most severe inborn errors of immunity (IEI). The impaired number or function of T cells, and often the associated defects in B and/or NK lymphocytes, result in increased susceptibility to lethal infections in infancy. Early diagnosis of TCL enables allogeneic hematopoietic stem cell transplantation (HSCT) or autologous gene-corrected therapy that can provide life-long correction of the immune defect. Most SCID babies are asymptomatic at birth, which emphasized the need for an assay that would rapidly and efficiently diagnose infants affected by profound TCL. Screening for TCL is now performed by quantifying T cell receptor excision circles (TREC) that are produced during the splicing and joining of the Variable, Diversity, and Joining gene segments in maturing thymocytes. The TREC episome remains in the cells that are released into the circulation, which can be quantified by PCR of dry blood spots collected on Guthrie cards. Importantly, TREC can serve as a surrogate marker for the presence of T cells in peripheral blood. NBS for TCL has dramatically changed the outcome of affected infants, prompting efforts to identify other IEI at birth [5, 6].

Humoral defects that impair production of immunoglobulins are considered the most common cause for symptomatic IEI [7]. Among these, profound B cell lymphopenia (BCL) including X-linked agammaglobulinemia (XLA), caused by defects in Bruton Tyrosine kinase, lead to markedly increased susceptibility to infections [8]. Infants with BCL are typically asymptomatic at birth. They are protected from infections in the first few months of life by maternal antibodies that crossed the placenta during pregnancy. However, when these antibodies degrade, typically around 4–6 months of age, or if insufficient antibodies passed during pregnancy, infants who are unable to produce their own antibodies will develop severe and frequently lethal infections [9]. These infections may also lead to irreversible organ damage, especially in the lungs. Administration of live viral vaccines in early childhood, such as oral polio, can lead to severe complications [10]. Later in life, chronic pulmonary disease, persistent gastrointestinal infections, septic arthritis and multiple bacterial infections may also occur [11]. XLA and other BCL, such as ataxia-telangiectasia or Nijmegen breakage syndrome, are also associated with increased risk for immune dysregulation and malignancies [12].

Many studies have documented significant diagnostic delays in XLA, with the time from symptom onset to diagnosis ranging from months to years [12, 13]. The diagnostic odyssey in XLA and other BCL has significant consequences as timely initiation of preventive treatments, such as antibiotic prophylaxis and immunoglobulin replacement therapy (IGRT), may prevent infections and improve the patients’ quality of life (QoL) [11, 14]. Early diagnosis and treatment might also prevent irreversible organ damage, allow better surveillance and targeted supportive care. The detection of index cases may further enable identification of undiagnosed family members. Additionally, identifying patients with BCL might improve the detection of “combined immune deficiencies” where both T and B cells are affected, such as Wiskott-Aldrich syndrome [15].

Given the importance of early diagnosis of patients with XLA and other BCL, diverse NBS strategies were investigated, including rapid multiplexed proteomic screening [16], targeted genetic testing [17] and Kappa light chain rearrangement excision circles (KREC) measurements [18]. KREC, produced during B cell development, analogous to the TREC in T cells, have generated the most interest [19]. In recent years, several studies have demonstrated the ability of KREC based NBS to detect XLA and other BCL, as well as their complementary role in diagnosing SCID and TCL with absent or reduced B cells. Some of these studies, each with more than 100,000 newborns screened, are detailed below and summarized in Table 1.

**Table 1:** The diagnostic yield of KREC newborn screening

Country	Number of newborns screened	Overall referred^#^	Reduced TREC and KREC referred^#^	Isolated reduced KREC referred^#^	Monogenic BCL identified^#^	PPV of reduced KREC for BCL^@^	Reference
Czech Republic	198,675	29	2	16.5	5	30.3%	20
Russia	202,908	46.5	3.5	4.5	0.5	11.1%	21
Russia	2,348,872	26	2.5	15	2	13.3%	22
Ukraine	398,415	14	5.8	6.3	0.25*	4%	23
Switzerland	435,985	23.6	NA	5.3	2	37.7%	24
China	103,240	113	1	69.8	2	2.8%	25

^#^per 100,000 newborns screened.

*Genetic testing not performed in all. NA- not available

^@^PPV- positive predictive value was calculated as the number of monogenic BCL identified divided by the number referred for isolated reduced KREC

A pilot TREC and KREC NBS program in the Czech Republic of 198,675 newborns led to the referral of 58 infants (0.029%), 21 due to low TREC and normal KREC, 33 with low KREC and normal TREC, and 4 with combined low TREC and KREC [20]. Amongst them, 10 patients with XLA and other BCL were identified (5/100,000 screened).

Similarly, a pilot study of 202,908 newborns from 8 Russian regions done in 2022 identified 191 with abnormal TREC and KREC results [21]. Following repeat testing, 93 were referred for immunological evaluations, including 77 with abnormal TREC, 7 for combined reduced TREC and KREC, and 9 with isolated reduced KREC. Genetic testing identified 3 patients with RAG1/2 and ADA deficiencies as a cause for T-B-SCID and 1 patient with IGLL1 deficiency. Notably, 16% of diagnosed newborns had a positive family history, including some with previously undiagnosed affected members, highlighting the value of early detection.

In 2023, the NBS program in Russia was expanded to include over 2.3 million newborns, becoming the largest report till now of combined TREC and KREC NBS [22]. Among these, 618 term and preterm infants were referred for flow cytometry analysis. Various forms of IEI were eventually diagnosed in 191 patients, including 37 with SCID and 47 with agammaglobulinemia, attributed to XLA and biallelic variants in the *IGLL1* gene (16 and 31 patients, respectively). Additional patients with reduced KREC were diagnosed with SCID and CID.

A Ukrainian program screened 398,415 newborns for reduced TREC and KREC, leading to the referral of 57 (0.01%) for confirmatory diagnostics, including 23 with reduced TREC and KREC and 25 with reduced KREC [23]. Genetic testing was not done in all children, limiting the ability to confirm monogenic IEI. Nevertheless, Nijmegen breakage syndrome and BTK defects were each diagnosed in 3 patients, while 4 more had autosomal recessive IGLL1 agammaglobulinemia.

Switzerland was one of the first countries to launch a nationwide TREC and KREC NBS program in 2019 [24]. Over the initial five years, 435,985 newborns were screened with an abnormal result rate of 0.09%. Abnormal TREC levels (with or without abnormal KREC), were found in 181 patients (47% of all patients with abnormal NBS results) while 205 (53%) had isolated reduced KREC levels. Repeated testing of the same Guthrie card limited the referral rate to 0.025%, with KREC accounting for only one-fifth of all referrals (0.005%). The program successfully identified 8 newborns with agammaglobulinemia and one patient with ataxia telangiectasia.

A study in Mainland China screened 103,240 newborns that resulted in referral of 72 infants (referral rate of 0.07%) and a diagnosis of 2 patients with XLA and one with Wiskott-Aldrich syndrome [25]. Overall, most KREC NBS programs maintained low referral rates while achieving high diagnostic yields, although the positive predictive value (PPV) for KREC was lower than TREC.

Cost-effectiveness was previously established for TREC NBS using various models and assumptions in many parts of the world [26]. A recent review of some of the studies indicated that universal NBS for SCID is likely to demonstrate health system and societal cost-effectiveness. The incremental cost-effectiveness ratio per quality-adjusted life-year (QALY) ranged from $30,214 to $54,282. In contrast to TREC, a possible cost-effectiveness of adding KREC to existing NBS has not been reported. Therefore, to assist in the decision to include KREC screening for XLA and other BCL in NBS programs, we aimed to develop an economic analysis similar to that established by the Jeffery Modell Foundation for SCID and related TCL disorders [27]. Accordingly, the objectives of the study were to develop an algorithm or ‘‘decision tree’’ to determine the role of KREC NBS for XLA and other BCL, based on peer-reviewed scientific literature on the prevalence and consequences of these conditions, their diagnosis and management. Another objective was to provide a transparent tool that could assist in determining the value of life in BCL while incorporating criteria and costs.

When determining the economic value for patients, family and the health care system of identification of XLA and other BCL shortly after birth, several factors were considered.

1. The incidence of XLA was previously estimated at 1 in 379,000 live births in the US [28], while other KREC-based NBS studies summarized in Table 1 suggest that the world-wide incidence of XLA and other BCL might be even higher and range between 1 and 5.7 among 100,000 live births.
2. The diagnosis of XLA is often delayed. The mean age of diagnosis of XLA in a large US registry was 5.4 years in patients with a negative family history [28]. Even with a positive family history, the mean age of diagnosis was 2.6 years, and only 34.5% of patients were diagnosed before clinical symptoms developed.
3. Infants with XLA and other BCL often suffer from recurrent infections and significant complications later in life, particularly chronic lung disease and bronchiectasis, reducing patients’ QoL and survival [29]. The increased morbidity and mortality can be prevented by early diagnosis and initiation of prophylactic antibiotics and IGRT [30]. Analysis of 231 patients with XLA enrolled in the USIDNET registry demonstrated that for every year of delayed treatment initiation, the odds of experiencing lower respiratory tract infection increased by 21.6% [31]. Similarly, a study focusing on lung disease in BCL found that initiation of IGRT significantly reduced the frequency of yearly lung infections in a patient with XLA from 1.67 to 0.45 episodes [32]. Thus, the diagnostic delay in XLA has significant clinical and financial consequences. Previous studies estimated that the healthcare cost prior to the diagnosis of humoral immune deficiency is $111,053 per patient/year [33]. The increased costs were attributed to frequent school/work absences (including parental), increased number of visits to health care providers and emergency departments, laboratory tests and imaging as well as hospitalizations [34].
4. IGRT cost varies by country and administration routes. In the US, Intravenous IGRT, usually in a supervised clinic setting, costs approximately $30,000 to $90,000/year, while in the UK, Canada, and Australia it is estimated to be less than $20,000/year [35]. Self-administered subcutaneous IGRT is substantially less costly than clinic administered intravenous IGRT [36].
5. The KREC NBS assay can be performed using the dried blood spot collected for other genetic disorders and can be combined with TREC, as both utilize mostly the same reagents, PCR methods, equipment and personnel. Some labs already use commercial assays for TREC that include KREC, such as the Food and Drug Agency approved multiplex PCR kit (https://www.revvity.com/category/newborn-screening-kits; https://www.immunoivd.com/products/trec-krec-screening-kit/), other commercial kits [22, 24], or “in-house” developed assays. Hence analyzing KREC using the existing TREC assay would have minimal expenses to the lab. Currently, the TREC-only assay is priced at $5 per sample [37]. We estimate that adding KREC would increase the cost of testing by 10%, hence an additional $0.5 per sample.
6. Referrals following positive screening were conceptually calculated as the number of monogenic BCL identified divided by the number isolated reduced KREC; because PPV varies widely across programs, we chose a conservative PPV of 14%, based on the cohorts summarized in Table 1, acknowledging that it is simple, unweighted average across heterogeneous programs and does not reflect study size. This implies roughly 12–15 referrals for every 2 true cases detected per 100,000 newborns. For simplicity of calculations, we determined that 14 newborns would be referred for the identification of 2 affected patients.

Based on these factors, the total cost and saving of adding KREC NBS for XLA and other BCL to an existing TREC NBS was calculated (Fig. 1). To simplify calculations, it was assumed that the screened population was of 100,000 newborns. Hence, KREC screening would cost an additional $50,000 ($0.5 × 100,000) and would identify 2 newborns with BCL. Using an assumed PPV of 14%, approximately 14 newborns with isolated reduced KREC would need to be referred for evaluation and flow cytometry to identify two patients with XLA or other BCL. The cost of analysis for abnormal KREC was estimated at $250 per patient [38], hence (14 referrals X $250) $3,500. Treating the 2 newborns with IGRT until 3 years of age, the average age that patients with XLA and BCL are identified, was estimated at $25,000/year/patient based on their weight (average weight of 10 kg) and average IGRT of $50,000 per year for an adult with XLA. Hence the total cost of IGRT for the 2 patients during the first 3 years of life would be $150,000, and the total cost of screening and treatment would be $203,500.

**Fig. 1:**
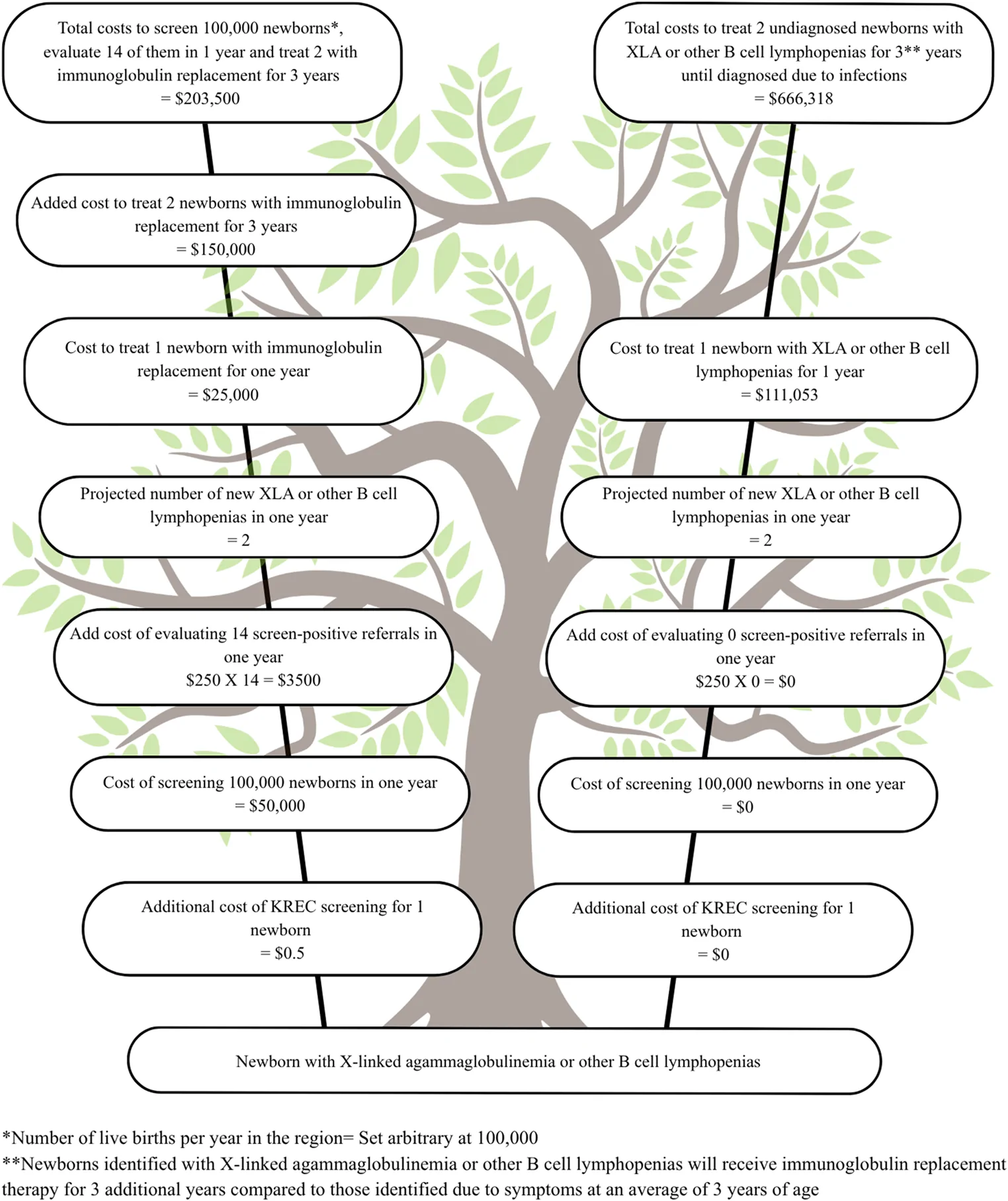
Costs estimate of KREC screening and treatment of X-linked agammaglobulinemia and other B cell deficiencies in comparison to the costs of non-screened newborns

The diagnosis of the 2 patients at birth, rather than at 3 years of age would significantly reduce the frequency of lung infections [32] and save medical expenses, estimated at $111,053 per patient/year [33], or $666,318 for both during this period. Additional indirect saving by the early diagnosis and intervention includes the prevention of chronic lung disease, which were previously shown to decrease the survival of patients with XLA at the age of 43 years from 98% to 90.5% [11]. Preventing the 7.5% mortality from 43 years of age to an average age of 70 years (27 years) with an estimated QALY of $27,907 per year [38] would amount to an additional financial benefit for the 2 patients of $113,023 (0.075 × 27 years X $27907/year X 2 patients). A registry study from the US reported 11.2% mortality at a median age of 21 years, suggesting an even greater financial benefit from early intervention [39].

In addition, we conducted deterministic one‑way sensitivity analyses varying each parameter over plausible ranges informed by published newborn screening programs and cost data. Ranges included were: incidence of XLA/other BCL 0.5-5/100,000; PPV of reduced KREC 2.8%-37.7%; incremental screening cost per newborn $0.25-$1.00; confirmatory testing cost per referral $150-$500; IGRT cost per patient‑year in infancy $15,000-$40,000/year; and annual healthcare costs avoided through earlier diagnosis $50,000-$150,000/year. The primary outcome was net short‑term savings to age 3 and a secondary outcome was net monetary benefit including monetized long‑term survival gains (QoL values $27,907, $50,000, and $100,000 in sensitivity analyses), with long‑term benefits discounted at 3% in a sensitivity analysis. Based on these ranges, we also examined best‑and worst‑case multi‑parameter scenarios. In our base case (incidence 2/100,000; PPV 10%), adding KREC to TREC screening cost $203,500 per 100,000 newborns and yielded $666,318 in healthcare costs avoided by age 3, for net short‑term savings of $462,818. Monetizing long‑term survival benefits added $113,023, for total net monetary benefit of $574,000 per 100,000 screened. Net short‑term savings remained positive across all ranges. In the best‑case scenario (high incidence/PPV, low costs, high treatment benefit), net short‑term savings were ≈ $2,000,000 while in the worst‑case scenario (low incidence/PPV, high costs, low benefit), the program incurred a net short‑term cost of ≈ $94,000, partially offset by long‑term monetized benefits. The short‑term break‑even incidence was ≈ 0.2 per 100,000. Parameters and costs for best case included: incidence 5/100,000; PPV 37.7%; screening $0.25; confirmatory $150; IGRT $15,000/yr; benefit $150,000/yr. Net short‑term savings ≈ $1,998,050 per 100,000. Parameters and costs for worst case included incidence 0.5/100,000; PPV 2.8%; screening $1.00; confirmatory $500; IGRT $40,000/yr; benefit $50,000/yr. Net short‑term savings ≈ $94,000 per 100,000.

XLA and other BCL, like many chronic ailments, significantly impairs the QoL of those affected. A self-reported questionnaire of European pediatric and adults suffering from XLA, and their parents, found that those diagnosed after 1 year of age had significantly reduced QoL in multiple health domains compared to the general population [30]. In contrast, patients diagnosed before 1 year of age reported improved QoL which was not different than the general population.

While previous studies showed the effectiveness of KREC NBS in detecting XLA and other BCL, and we show here its financial benefits, several additional factors should be considered. Patients with XLA and other BCL may have a small number of residual B cells in their blood [40]. Therefore, striking a balance between maximizing detection rates and minimizing harm is critical, underscoring the importance of optimizing screening algorithms. For example, revising the cut-off values of TREC and KREC in the Swiss study reduced the number of abnormal screening results [24], which also has major financial implications. This also emphasizes that some patients might still not be diagnosed through NBS, requiring continued vigilance among health care providers for the possibility of XLA and other BCL presenting at a later age.

Although IGRT is effective in protecting against many bacterial and viral pathogens, the increased susceptibility to infections and non-infectious complications in patients with XLA and other BCL is not entirely averted by antibiotics and IGRT [41]. Therefore, the financial benefits of NBS might be lower than originally calculated. Nevertheless, appreciating that the patient with these complications suffers from IEI may lead to quicker and more efficient management, contributing to cost savings.

The financial benefits of NBS for XLA and other BCL might be even more pronounced as treatments become less expensive, either because of the reduction in the costs of IGRT or the availability of innovative management options. For example, HSCT was recently shown to be 2–3 times cheaper than life-long IGRT [35]. Moreover, the possibility of autologous gene therapy for XLA, which might have less complications than allogeneic HSCT, is currently being investigated [42].

Our study has several notable limitations that may affect the cost-effective analysis. We used a simplified cost‑offset (budget‑impact) approach rather than a full comparator‑based cost‑effectiveness model: we did not pre‑specify an analytic perspective or willingness‑to‑pay threshold or model health outcomes. The time horizon was short (birth to typical age of diagnosis) and did not apply discounting; long‑term benefits were summarized as point estimates, so results should be viewed as indicative of potential short‑term cost offsets rather than definitive evidence of cost‑effectiveness. Our base‑case referrals were derived using a conservative, midrange PPV assumption that likely overstates confirmatory testing volumes and costs; using a pooled PPV would reduce referrals and modestly strengthen the economic case. We also implicitly treated much pre‑diagnosis utilization as avoidable with newborn detection and included screening, confirmatory testing, and early IGRT costs but did not explicitly model ongoing post‑diagnosis costs in the screened arm or those accruing pre‑diagnosis in the unscreened arm, making short‑term savings an upper bound. Finally, we combined clinically heterogeneous KREC‑detectable disorders while applying assumptions largely informed by XLA. Other KREC‑detectable disorders (e.g., ataxia‑telangiectasia, Nijmegen breakage syndrome, some combined immunodeficiencies) differ in natural history, management, and the profile of benefits from early detection (e.g., vaccine/radiation avoidance, oncology surveillance, targeted prophylaxis), which may reduce short‑term cost offsets compared with XLA while introducing different long‑term benefits not captured here. Future work should standardize PPV estimation (e.g., weighted pooling), derive referrals directly from the chosen PPV, and compare total costs for screened versus unscreened pathways in a full decision‑analytic model.

In conclusion, KREC-based NBS can lead to early diagnosis and treatment of XLA and other BCL, preventing short- and long-term morbidity and mortality, and is also likely cost‑saving over the early diagnostic‑delay period under our base‑case assumptions. The KREC screening assay fulfills the Wilson and Junger criteria, therefore it should be included in NBS.

## Data Availability

No datasets were generated or analysed during the current study.

## Abbreviations

BCL: B cell lymphopenia
HSCT: Hematopoietic stem cell transplantation
IEI: Inborn errors of immunity
IGRT: Immunoglobulin replacement therapy
KREC: Kappa light chain rearrangement excision circles
NBS: Newborn screening
PPV: Positive predictive value
QALY: quality-adjusted life-year
QoL: Quality of life
SCID: Severe combined immunodeficiency
TCL: T cell lymphopenia
TREC: T cell receptor excision circles
XLA: X-linked agammaglobulinemia
